# Decomposition and organic amendments chemistry explain contrasting effects on plant growth promotion and suppression of *Rhizoctonia solani* damping off

**DOI:** 10.1371/journal.pone.0230925

**Published:** 2020-04-09

**Authors:** Giuliano Bonanomi, Maurizio Zotti, Mohamed Idbella, Nice Di Silverio, Linda Carrino, Gaspare Cesarano, Abdulaziz M. Assaeed, Ahmed M. Abd-ElGawad

**Affiliations:** 1 Department of Agricultural Sciences, University of Naples Federico II, Portici, Naples, Italy; 2 Department of Biology, Faculty of Sciences and Techniques, Hassan II University, Mohammedia, Morocco; 3 Plant Production Department, College of Food & Agriculture Sciences, King Saud University, Riyadh, Saudi Arabia; 4 Department of Botany, Faculty of Sciences, Mansoura University, Mansoura, Egypt; Sichuan Agricultural University, CHINA

## Abstract

Organic Amendments (OAs) has been used in agroecosystems to promote plant growth and control diseases caused by soilborne pathogens. However, the role of OAs chemistry and decomposition time on plant growth promotion and disease suppression is still poorly explored. In this work, we studied the effect of 14 OAs at four decomposition ages (3, 30, 100, and 300 days) on the plant—pathogen system *Lactuca sativa*–*Rhizoctonia solani*. OAs chemistry was characterized via ^13^C-CPMAS NMR spectroscopy as well as for standard chemical (i.e. N content, pH, EC) and biological parameters (i.e. phytotoxicity and *R*. *solani* proliferation bioassay). OAs have shown variable effects, ranging from inhibition to stimulation of *Lactuca sativa* and *Lepidium sativum* growth. We recorded that N rich OAs with high decomposability were conducive in the short-term, while converting suppressive in the long term (300 days). On the other hand, cellulose-rich OAs with high C/N ratio impaired *L*. *sativa* growth but were more consistent in providing protection from damping-off, although this property has significantly shifted during decomposition time. These results, for the first time, highlight a consistent trade-off between plant growth promotion and disease control capability of OAs. Finally, we found that OAs effects on growth promotion and disease protection can be hardly predictable based on the chemical characteristic, although N content and some ^13^C CPMAS NMR regions (alkyl C, methoxyl C, and carbonyl C) showed some significant correlations. Therefore, further investigations are needed to identify the mechanism(s) behind the observed suppressive and conducive effects and to identify OAs types and application timing that optimize plant productivity and disease suppression in different agro-ecosystems.

## 1. Introduction

Hystorically, the use of Organic Amendments (OAs) has been considered a recommended practice in conventional and organic agriculture. Addition of OAs in agricultural soil, enhance soil fertility and structure [1,2] and, moreover, protect crops by controlling diseases caused by soilborne pathogens [3,4]. The introduction of synthetic fertilizers over the past centuries encouraged farmers to reduce the practice of OAs application [5] which in turns, limited the crucial role of OAs as soilborne pathogen controlling agents. As consequence, in modern agriculture, the lack of suppressiveness from OAs bring to a dependencies by synthetic products for pests control. Nowadays, the public concerns about the effect of synthetic fertilizers, herbicides and fungicides on human health and environment resulted in the restriction of soil fumigants and fungicides that however was the most effective way to control soilborne pathogens. Moreover, fungicide resistance in pathogens and the unreliability of chemical control have contributed to the development of a variety of alternative control measures [6].

OAs play different roles in plant disease control. On the one hand by enhancing the activities of newly introduced biocontrol agents [4], and on the other hand, by encouraging the natural suppression of plant pathogens by supporting the native soil microbiota [7]. OAs potential to suppress disease is mostly pathogen-specific and varies widely between pathogen types. Observations have shown a reliable control of *Verticillium dahliae* [8], *Thielaviopsis basicola* [9], several species of *Fusarium* [10], *Phytophthora* [11], and *Pythium* [12]. However, the control of *Rhizoctonia solani* using OAs seems to be more difficult with a literature documentation stating only 26% of application among all cases [13]. *R*. *solani* can survive in the soil for several years in the form of sclerotia or as active mycelium. In fact, *R*. *solani* can thrive as a saprophyte in soil thanks to its capability to degrade simple sugar as well as cellulose and hemicellulose. In general, this fungus can survive in soil even in absence of plant host because of the capability to effectively exploit a larger range of OA types [14,15,16]. Moreover, when agricultural soil is amended by adding organic residues with a high C:N ratio, the competition for nitrogen will decrease the saprophytic activity of *R*. *solani* and in turn its potential pathogenicity [17].

The introduction of OAs to soil or potting substrate triggers a decomposition process that is related to factors like temperature, humidity, and organic matter quality [18]. The interconnection of these variables makes it difficult for the prediction of the effect of OAs decomposition on soil pathogens suppression. Thereafter, it is important to analyze and characterize the OAs to better understand their quality, chemistry, and changes that occur during their decomposition. In this regards, solid-state ^13^C-CPMAS NMR is a useful tool to analyze OAs quality [19] additionally to the conventional chemical analysis, as total C and N content. In fact, from the farmer’s point of view, it is important to understand and predict the impact of amendment application on both plant growth and disease suppression. The degree of OAs decomposition greatly affects, positively or negatively, plant growth [20,21] as well as pathogens suppression capability [22,5]. Changes in the suppressive capability of OAs and level of decomposition have been described in different studies for organic wastes [15], peats [23], and composts [24]. For instance, mature composts were found to be suppressive of *R*. *solani* damping-off, while incompletely decomposed materials intensified it [25].

The contrasting results obtained in disease control experiments with OAs that have shown both suppressive and conducive effect indicate the need for further researches. In particular, it is important to address the reliability of methods for predicting the effect of different OAs on soilborne pathogens and balancing the suppressive ability of OAs with respect to pathogens and to plant growth and productivity. For example, among soilborne pathogens, *R*. *solani* is one of the most studied but few appreciable results of its suppression have been demonstrated via the use of OAs [26,27]. In this context, the aim of this study is to explore the link between OAs chemistry, with two agro-ecosystem functions: plant growth promotion and diseases suppression. We used 14 organic amendments at different decomposition ages (3, 30, 100, and 300 days) on the plant—pathogen system *Lactuca sativa*–*R*. *solani*. OAs chemistry was characterized via ^13^C-CPMAS NMR spectroscopy as well for standard chemical parameters. Specific aims of this work were:

- Assessment of the effect of different OAs and decomposition time on either plant growth promotion or suppression of *R*. *solani*;
- Explore the relationships between OAs chemistry, as defined by ^13^C-CPMAS NMR spectroscopy, with plant growth and disease suppression;
- Search for chemical parameters able to distinguish suppressive from conducive OAs.

## 2. Materials & methods

### 2.1 Organic amendments and chemical analyses

Fourteen OA types were selected: (1) composted green amendment (compost pam); (2) organic fraction of municipal bio-waste (FORSU); (3) dried and finely crushed meat residue; (4) pelleted fish feed; (5) *Medicago sativa* hay; (6) *Zea mays* residues; (7) biochar obtained from sawdust pyrolized at 300°C; (8) biochar obtained from sawdust pyrolyzed at a temperature of 550°C; (9) coconut fiber cultivation substrate; (10) sphagnum peat; (11) concentrate of humic acids; (12) sawdust; (13) glucose, (14) and cellulose. All materials were dried, ground and sieved using a 2 mm mesh size. OAs were chemically characterized for total C and N content by flash combustion of micro samples (5 mg of sample in an Elemental Analyzer NA 1500 Fison 1108 Elemental Analyzer, Thermo Fisher Scientific). The pH and EC values of OAs aqueous suspensions were measured using a pH-meter and a conductimeter, respectively. OAs, moreover, were analysed by solid state ^13^C-CPMAS NMR in order to obtain a detailed comparison of molecular properties. The NMR spectra were obtained by Bruker AV-300 NMR spectrometer (Bruker Instrumental Inc, Billerica, MA, USA), equipped with a magic angle spinning (MAS) probe with widebore of 4 mm, using specific calibrated acquisition features: 2 s of recycle time; 1H-power for CP 92.16 W: 1H 90° pulse 2.85 μs; ^13^C power for CP 150,4 W; 1 ms of contact time; 20 ms of acquisition time; 2000 scans. The spectral regions were selected and identified in the corresponding classes of organic C bonds as described in previous studies [19,28]. The seven chemical shift regions representative of main C types were considered with restricted regions of relevance in brackets (Table 1).

**Table 1:** The seven chemical shifts regions given in ppm by NMR and their correspondent classes of C types.

Chemical shift (ppm)	Correspondent C types
0–45	alkyl + alpha amino C
46–60	N-alkyl C (56 ppm = methoxyl, alpha-amino)
61–90	O-alkyl C
91–110	di-O-alkyl C (103–105 ppm = anomeric C in carbohydrate, quaternary aromatic carbons in tannins)
111–140	H and C- substituted aromatic C (126 ppm = unsubstituted)
141–160	O-substituted aromatic C (phenolic and O-aryl C, 147–153 ppm = heterosubstituted, vanillyl + syringil lignin units)
161–190	carbonyl C (172 ppm = carboxyl + amide, 198 ppm = ketone/aldehyde)

The relative contribution for the specific region was assessed by the integration of MestreNova 6.2 software (Mestre-lab Research 2010), and expressed as a percentage of the total area.

Three indices of OAs decomposition state were calculated: (i) the O-alkyl C / methoxyl and N-alkyl C ratio (thereafter indicated as CC/MC); (ii) the alkyl C / O-alkyl C ratio [29,30]. In addition, a new index proposed by Bonanomi et al. [31] was calculated as the ratio between two restricted regions of O-alkyl C (70–75 ppm) and methoxyl C (52–57 ppm), respectively (i.e. 70-75/52-57).

### 2.2 Organic amendments phytotoxicity

The experiment was conducted to evaluate the phytotoxicity of the fourteen organic amendments on two plant species. Plant susceptibility to OAs phytotoxicity was measured through root length parmaters because of the increased sensitivity of roots to aqueous extracts compared to germination [32]. *L*. *sativa* and *Lepidium sativum* have been selected as experimental crops because of their rapid and uniform germination. For each OA, an aqueous extract was made by adding 400 ml of D-H_2_O to each 2 g of dry material. The suspension was placed in an electric shaker for 5 hours under a controlled temperature of 25°C in order to promote outflow of plant biochemical constituents. The extracts obtained were filtered with filter paper and then stored in a freezer at –20°C. The phytotoxicity test was carried out with three different concentrations, for all the 14 materials: raw material (corresponding to 5% dry matter suspended in water); 1:3 (1.5% dry matter) and 1:10 (0.5% dry matter). In Petri dish (Ø: 9 cm), four ml of each suspension was added along with ten surface sterilized seeds of each plant species. Control was made up with the same process but with adding D-H_2_O in Petri dish instead of OA extract. The experiment lasted for 72 and 144 hours for *L*. *sativum* and *L*. *sativa*, respectively. At the end of the experiment, root length was measured. Overall, the experiment follows a full factorial design with 14 OAs types at three concentrations. Trial was carried out in 5 replicates for two plant species. The experiment ends up with a total of 430 experimental units and 4,300 seeds (14 OAs x 3 Concentrations x 5 Replicates x 2 Plant species plus 2 Plants species as control x 5 Replicates for a total of 430 experimental units).

### 2.3 Organic amendments effect on *R*. *solani*

A hyphal growth experiment was performed to assess the effects of the 14 undecomposed OAs on the saprotrophic growth capability of *R*. *solani*. In order to assess only the effect of OAs as carbon and nutrient source, the fungal growth was initiated from oligothorphic medium, water agar (WA, Oxoid). After seven days of fungal growth over WA, a 3 mm diameter plug was transferred from the periphery of the colony to a new petri dish containing 15 ml of a substrate comprising: 1 part of WA and 1 part of sterile organic extracts (filter sterilized at 0.22 μm). WA was used as the control substrate. Each treatment was replicated five times. After 72 hours, four random points were selected to measure the hyphal length and density. Measurement was made by means of an optical microscope at 250 x magnification, Hyphal density parameters was determined by counting the number of hyphae that cross a 1 mm orthogonal line. Following, growth index representative of the response of the *R*. *solani* to OAs extract addition was calculated as the product of colony radius and hyphal density [25].

### 2.4 Plant growth promotion and damping-off bioassay

Suppressiveness of the 14 OAs was assessed by measuring the disease incidence and severity in *L*. *sativa*–*R*. *solani* AG-4 HGII pathosystem. *R*. *solani* isolates were obtained from lettuce seedlings [33] and the determination of anastomosis group was done according to Dong et al. [34].

The experiment was fully factorial combining OAs (14 types), incubation time (3, 30, 100, and 300 days), and presence or absence of *R*. *solani* inoculum. Each treatment was replicated five times, for 600 pots including the control without organic amendment. Plant growth experiment was conducted in pots with 12 cm diameter and 10 cm height. Sand-vermiculite mixture in the 4:1 ratio in an amount of 250 g for each pot. Following the experimental design, each pot was used as a growing substrate assigned a treatment and was amended with 2% in dry weight (w/w) of a respective OAs and incubated for 3, 30, 100 or 360 days in a growth chamber under controlled temperature (22±2°C during the day and 18±2°C at night) and irrigated every 7 days to field capacity. At the end of the incubation period, pots were moved to a greenhouse where they were inoculated with the pathogen.

*R*. *solani* inoculum was prepared using 300 g of common millet that was placed in 250 ml flasks, saturated with potato dextrose broth solution (1/10). The material was autoclaved and inoculated with *R*. *solani* plug collected from a potato dextrose agar plate and, thereafter, the flasks were incubated for 18 days at 20°C. The *R*. *solani* inoculum was dried for 5 days in sterile conditions, powdered and applied up to 0.1% (dry weight) to pots. In the not inoculated control, only common millet was added to pot mixture. Four days after *R*. *solani* inoculation, each pot was planted with ten lettuce seeds (fungicide untreated seeds cv. Chiara). The experiment was carried out in a greenhouse (24±5°C during the day and 16±5°C at night) and the pots were arranged following a completely random design, with rotation that was done every week. The pots were irrigated with distilled water every two days up to water holding capacity and saucers were used under the pot in order to prevent carry-over of inoculum. Every seven days during the experiment, the number of plants alive, dead, and attacked by *R*. *solani* in each pot was counted. At the end of the experiment, after 40 growing days, plant shoots were harvested, washed with tap water and dry weighted (80°C until a constant weight was reached).

### 2.5 Data analysis

The bioassay was carried out with a soil type inoculated with *R*. *solani* or without, with 14 OAs at five decomposition stages for 70 amended types. The effect of OA type and decomposition time, in absence of inoculum, on lettuce growth was assessed by two-way ANOVA using *L*. *sativa* dry weight. One-way ANOVA was used to assess the effect of OAs type on *R*. *solani* growth over sterile extract of the organic materials. The software STATISTICA 7 (StatSoft Inc., USA) and R was used for statistical analyses.

*R*. *solani* impact on seedling and plant survival was quantified with the survival index (SI) calculated as follows:

$$SI=\left(P_{T}/P_{C}\right)\;x\;100$$

Where *P*_*T*_ is the number of alive plants in pots with inoculum and *P*_*C*_ is the number of alive plants in pots without inoculum. The survival index was calculated with data recorded 28 days after sowing. In order to describe the chemical diversity of the 14 OAs, a data matrix derived from ^13^C-CPMAS NMR spectra was submitted to Cluster Analysis. The complete linkage based on Pearson’s correlation coefficient was used as a similarity parameter. To explore the association between the OAs chemistry with plant growth and disease suppression, simple correlation analysis was carried out with elemental chemical parameters (i.e. C and N content, C/N ratio, pH, EC) and specific ^13^C-CPMAS NMR regions selected from literature [30,28]. Statistical correlation significance was controlled for multiple comparisons according to Bonferroni’s correction. Finally, ^13^C-CPMAS NMR data were submitted to Principal Component Analysis (PCA), in order to provide a synthetic representation of OAs chemistry and their effects on *L*. *sativa* growth and disease suppression. In detail, we used the approach proposed by Legendre and Legendre. [35] for supplementary variables (i.e. plant growth and disease suppression) that were plotted as loading vector in the bi-dimensional PCA space, but not used to compute the eigenvalues of the ordination space. Pearson’s correlation coefficient, cluster analysis, and PCA were carried out using the software XLSTAT (version 2018, Addinsoft, NY, USA, www.xlstat.com)

## 3. Results

### 3.1 Organic amendments chemistry

**Fig 1:**
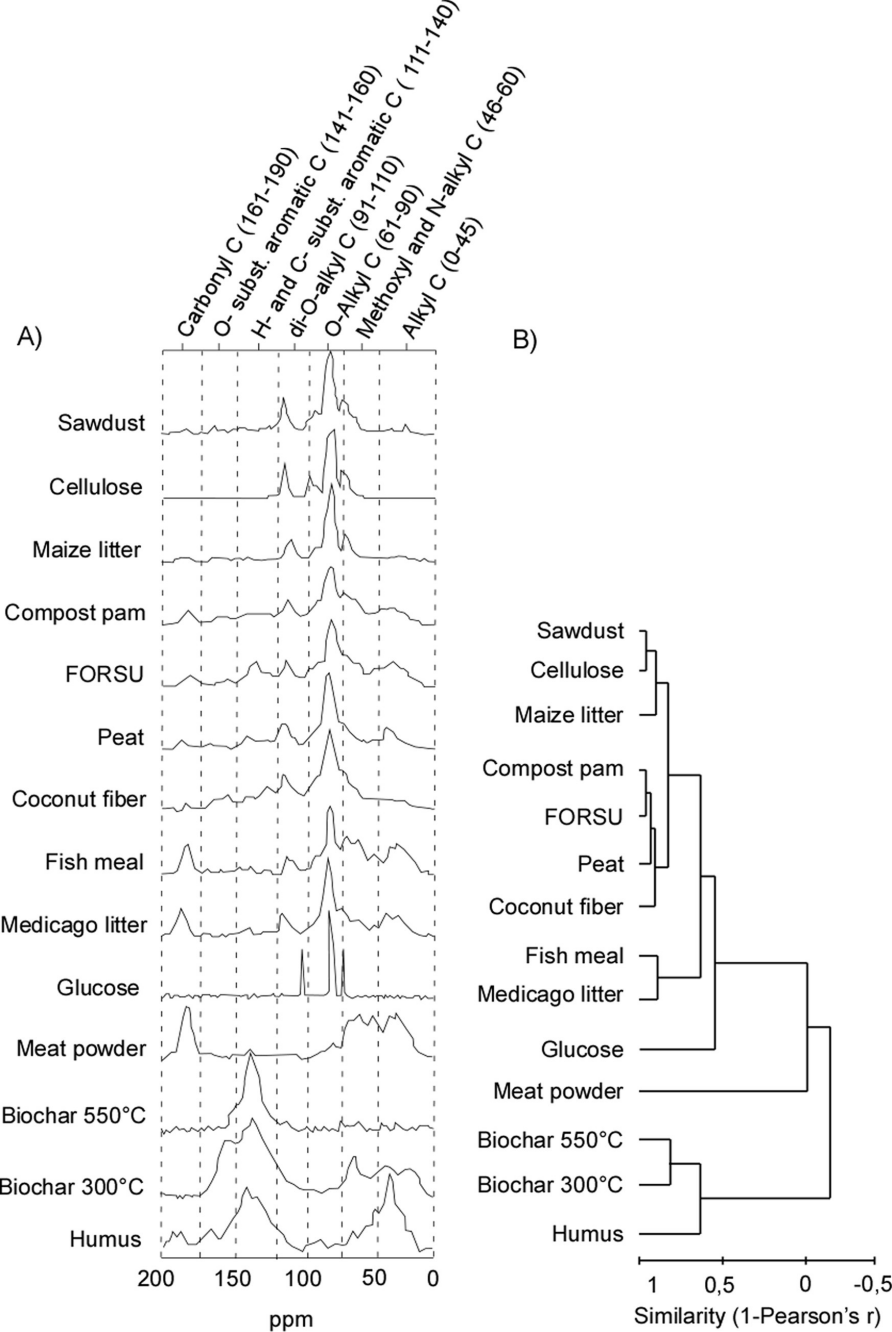
Chemical differences among organic materials, used for soil amendment. (A) ^13^C-CPMAS NMR spectra of the materials. Reference spectral regions and corresponding C types are reported on top of the panels, with chemical shift ranges indicated in brackets and by vertical dotted lines. (B) Dendrogram of organic materials based on spectral data.

The 14 OAs showed notable differences in chemical composition in terms of element content, and molecular composition as quantified by ^13^C-CPMAS NMR spectra (Table 2, Fig 1A). Fish meal, meat powder, and *Medicago* litter had very high N content and electrical conductivity, with a correspondingly low C/N ratio (Table 2). On the contrary, compared to *Zea* and in particular to wood feedstocks glucose, cellulose, sawdust, coconut fiber and maize litter having low N content resulting in low C/N ratio, in all cases with values higher than 75. In this regard, the two biochars have exceptionally high C/N ratio values (>400) determined by the very high C concentration combined with their low N content. In addition, pH has shown a large variation, ranging from 9.4 of biochar pyrolized at 550°C to 4.6 of peat. EC values was very high for humus, compost, and *Medicago* litter, while low values were found for the two biochars, glucose and cellulose.

**Table 2:** Content of carbon, nitrogen, pH, electric conductivity, C/N and ^13^C CPMAS NMR data of the 14 organic amendments.

Parameters	Biochar 300°C	Biochar 550°C	Cellulose	Coconut fiber	Compost pam	Fish meal	FORSU	Glucose	Humus	Maize litter	Meat powder	Medicago litter	Peat	Sawdust
**Chemical**														
C %	85.59	87.71	50.00	37.50	31.00	74.19	45.00	43.0	35.90	40.38	43.88	38.29	42.00	49.88
N %	0.13	0.21	0.10	0.50	1.52	6.06	1.91	-	2.40	0.49	8.26	3.93	0.60	0.11
C/N ratio	658.15	417.67	500.00	75.00	20.39	12.24	23.56	-	14.96	82.40	5.31	9.74	70.00	453.45
pH	4.93	9.38	7.06	4.81	6.37	6.25	9.24	6.61	6.30	7.05	5.82	5.82	4.60	5.70
EC mS/cm	0.08	0.15	0.13	2.85	3.18	1.29	1.96	0.02	4.01	2.65	2.21	3.45	0.03	0.42
^**13**^**C-CPMAS NMR**														
Carbonyl-C (161–190 ppm)	1.72	4.08	0.25	1.75	7.06	9.34	5.29	4.24	8.42	2.63	19.54	11.15	5.04	2.97
O-subst. aromatic C (141–160 ppm)	13.00	5.23	0.75	6.27	5.75	3.00	3.23	4.40	8.94	1.21	2.53	2.01	4.03	3.56
H-C subst. aromatic C (111–140 ppm)	39.08	65.38	2.28	13.44	12.31	5.93	14.12	6.70	33.17	2.93	4.47	6.46	10.58	7.64
di-*O*-alkyl C (91–110 ppm)	8.87	5.98	16.90	15.61	11.32	6.00	10.22	15.84	4.94	14.16	1.90	8.75	12.42	14.50
*O*-alkyl C (61–90 ppm)	5.69	5.29	74.79	44.61	34.54	31.59	36.23	61.06	4.76	61.07	8.94	38.53	42.08	56.91
Methoxyl C (46–60 ppm)	9.27	4.49	3.77	9.04	11.63	16.32	10.44	2.34	6.20	8.39	20.99	10.13	8.12	8.13
Alkyl C (0–45 ppm)	22.37	9.55	1.26	9.29	17.39	27.82	20.48	5.41	33.56	9.61	41.61	22.97	17.74	6.30
^**13**^**C-CPMAS NMR ratios**														
Alkyl C/*O*-alkyl C	1.54	0.85	0.01	0.15	0.38	0.74	0.44	0.07	3.46	0.13	3.84	0.49	0.33	0.09
CC/MC	0.61	1.18	19.86	4.94	2.97	1.94	3.47	26.09	0.77	7.28	0.43	3.81	5.18	7.00
70-75/52-57 ppm	0.23	0.47	32.83	3.77	2.10	1.71	2.76	64.07	0.24	11.96	0.25	3.80	5.20	6.5

Considering C bond types derived from NMR spectrum, the alkyl-C (0–45 ppm), the methoxyl and *N*-alkyl C (46–60 ppm) fractions showed high peaks in meat powder, fish meal, *Medicago* litter, FORSU and biochar made at 300°C (Table 2; Fig 1A). The di-*O*-alkyl-C (91–110 ppm) and *O*-alkyl-C (61–90 ppm) regions that are associated with polysaccharides and sugars were especially high in glucose, cellulose, maize litter, sawdust, and coconut fiber. The *H*- and *C*-substituted aromatic C (111–140 ppm) fraction was very abundant in the two biochar and for humus. The *O*-substituted aromatic C fraction (141–160 ppm) was not abundant in the studied OAs, with the exceptions of the two biochar and humus. Finally, the carboxylic C (161–190 ppm) was abundant in meat powder, fish meal, and, to a lesser extent, in *Medicago* litter.

The cluster analysis (Fig 1B), based on spectral signals and related C bond types, allowed a similarity comparison among the 14 OAs. The two biochar and humus grouped in a separate way from all other OAs. Another distinctive segregation is notable for glucose and meat powder showing dissimilarity from other OAs. Higher level of similarities is detected for the other OAs analyzed with a specified level of segregation for more similar *Medicago* litter and fish meal, for compost pam, coconut fiber, peat, and FORSU forming an intermediate group and, lastly, a more dissimilar group formed between sawdust, cellulose and maize litter.

### 3.2 Organic amendments phytotoxicity

**Fig 2:**
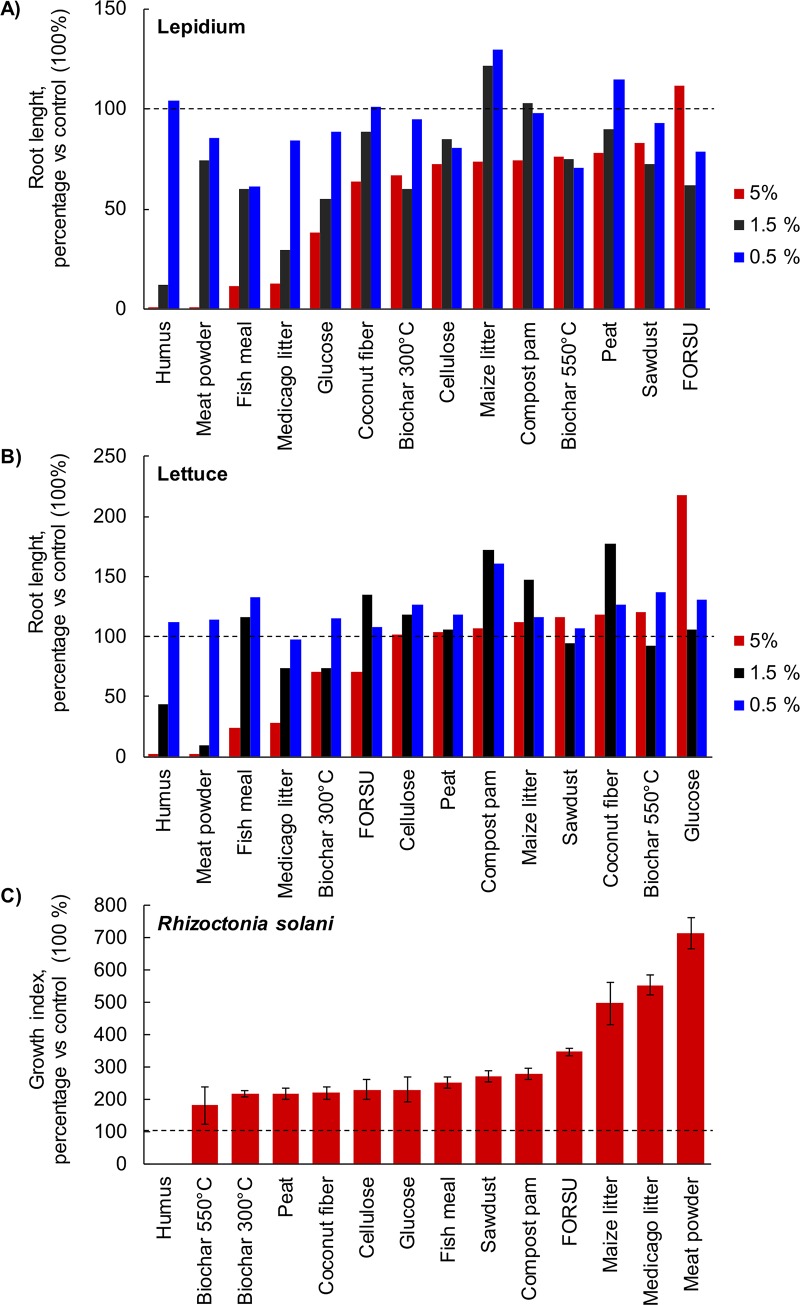
Responses of Lepidium sativum (A) and *Lactuca sativa* (B) to watery extracts (50, 5, and 0.5 g l^-1^) of organic amendments. Data refer to root length, normalized to control plants. The bars represent the standard deviation.

The bioassay concerning the effects of the 14 OAs water extracts on plant germination and root growth showed quite similar results for *L*. *sativa* and *L*. *sativum* (Fig 2). There was strong inhibition, that was significantly dependent by extract concentration, for meat powder, fish meal, humus, and *Medicago* litter. Other OAs showed minor but significant root growth inhibition only at the highest extract concentration. On the other hand, maize litter promoted root growth of *L*. *sativum*, whereas glucose, maize litter, coconut fiber, and FORSU promoted root growth of *L*. *sativa* compared to untreated control.

### 3.3 Organic amendments effect on *R*. *solani* growth

The *R*. *solani* growth index was significantly affected by OA type (S2 Table). *R*. *solani* was inhibited only by humus extract, while the growth was similarly with biochar, peat, coconut fiber, cellulose, glucose, fish meal, sawdust, and compost pam (Fig 2C). The growth index was especially high in presence of on FORSU, maize litter, *Medicago* litter, and meat powder.

### 3.4 Organic amendments and plant growth promotion

**Fig 3:**
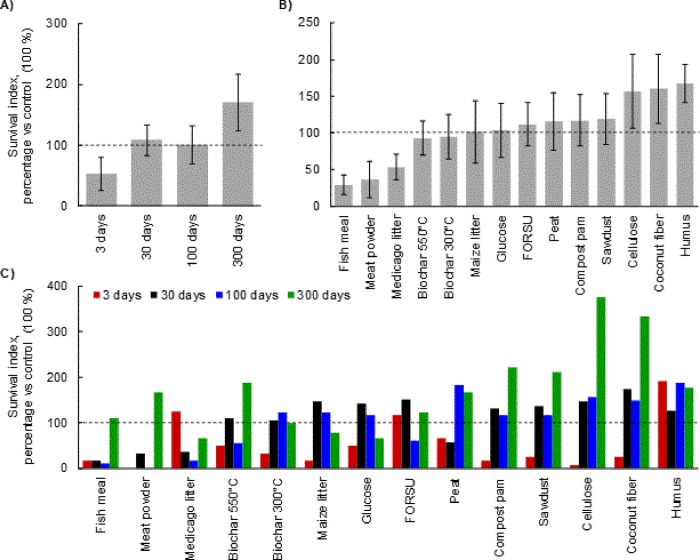
Lettuce growth. Data refer to the weight of *Lactuca sativa*, normalized to control plants, as average for all materials at each time of decomposition (3,30, 100 and 300 days) (A) and as average in all time of decomposition for each material (B). In (C) the weight is represented for each material and for each decomposition date. The bars represent the standard deviation.

In the absence of *R*. *solani*, plant growth was significantly affected by OA type and decomposition time (Fig 3). *L*. *sativa* response to the various OAs showed a large variability, depending on the decomposition stage. Overall, as OAs decomposition age increases, we observed an enhanced growth promotion effect (Fig 3A). When data from all decomposition times were pooled, *L*. *sativa* growth was inhibited by glucose, cellulose, maize litter, humus, and sawdust; however, peat and biochar 550°C showed no effect, while the remaining materials demonstrated a growth promotion effect, especially compost pam, fish meal, and meat powder. When all decomposition data were considered separately, the trends observed during decomposition were divided into four categories: increased growth, constant growth, decreased growth, and decreased growth followed by an increase (indicated as U-shaped). Five OAs (i.e. FORSU, *Medicago* litter, fish meal, meat powder, and biochar 300°C) showed strong growth inhibition in early stages (i.e. 3 days), that turns into a progressive growth recovery which, in some cases, resulted in a highly significant growth promotion (Fig 3C). Inversely, sawdust amendment reduced *L*. *sativa* growth, with an inhibitory effect that intensifies as decomposition proceeded. The U-shaped response was observed for biochar 550°C, coconut fiber, glucose, cellulose, humus, and maize litter. Finally, decomposition time did not affect the *L*. *sativa* response to peat and compost pam amendments.

### 3.5 Organic amendments and *Rhizoctonia* damping-off

**Fig 4:**
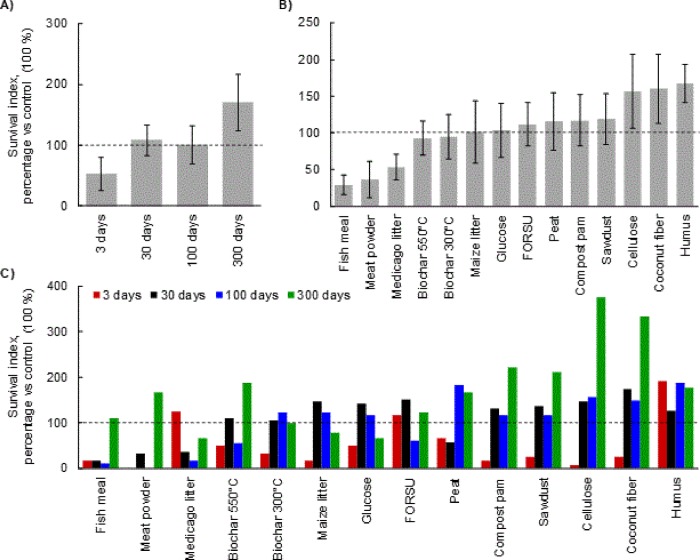
*Lactuca sativa* survival index (SI) in presence of Rhizoctonia solani inoculum. In (A) the SI is expressed as average for all materials at different time of decomposition (3, 30, 100 and 300 days), in (B) as average at all time of decomposition for different organic material. In (C) the survival index is reported for each material at all decomposition time. The bars represent the standard deviation, for statistical details see S4 Table.

OA types, decomposition time, and their interaction significantly affected the SI of *Rhizoctonia* (S4 Table). When data from all OAs were pooled, we observed a lower SI at the early decomposition stage (i.e. 3 days), similar to control after 30 and 100 days of incubation, with the highest value at the later decomposition stage (i.e. 30 and 300 days) (Fig 4A). Noteworthy, only in three cases (21%), OAs were suppressive after three days of incubation while these values rose up to ten (71%) after 300 days of decomposition. When data from all decomposition time were pooled, the SI was lower than control (i.e. conducive effect) for fish meal, meat powder, and *Medicago* litter (Fig 4A). The SI was comparable to control for biochar, glucose, peat, FORSU, compost pam, maize litter, and sawdust, while a suppressive effect (i.e. positive SI) was found for cellulose, coconut fiber, and humus.

When all decomposition data and OAs were considered separately, we found four main trends: increased disease suppression, constant disease suppression, decreased suppression followed by an increase (indicated as U-shaped), and increased suppression followed by a decrease (indicated as Ո-shaped). Nine OAs (i.e. fish meal, meat powder, the two biochars, peat, compost pam, sawdust, cellulose, and coconut fiber) were conducive in the early decay phases, but showed a progressive increase of the SI (Fig 4C). The U-shaped response was observed for *Medicago* litter and FORSU, while the Ո-shaped response was recorded for maize litter and glucose. Finally, only humus was consistently suppressive.

### 3.6 Relationships between plant growth, disease suppression, and organic matter chemistry

**Fig 5:**
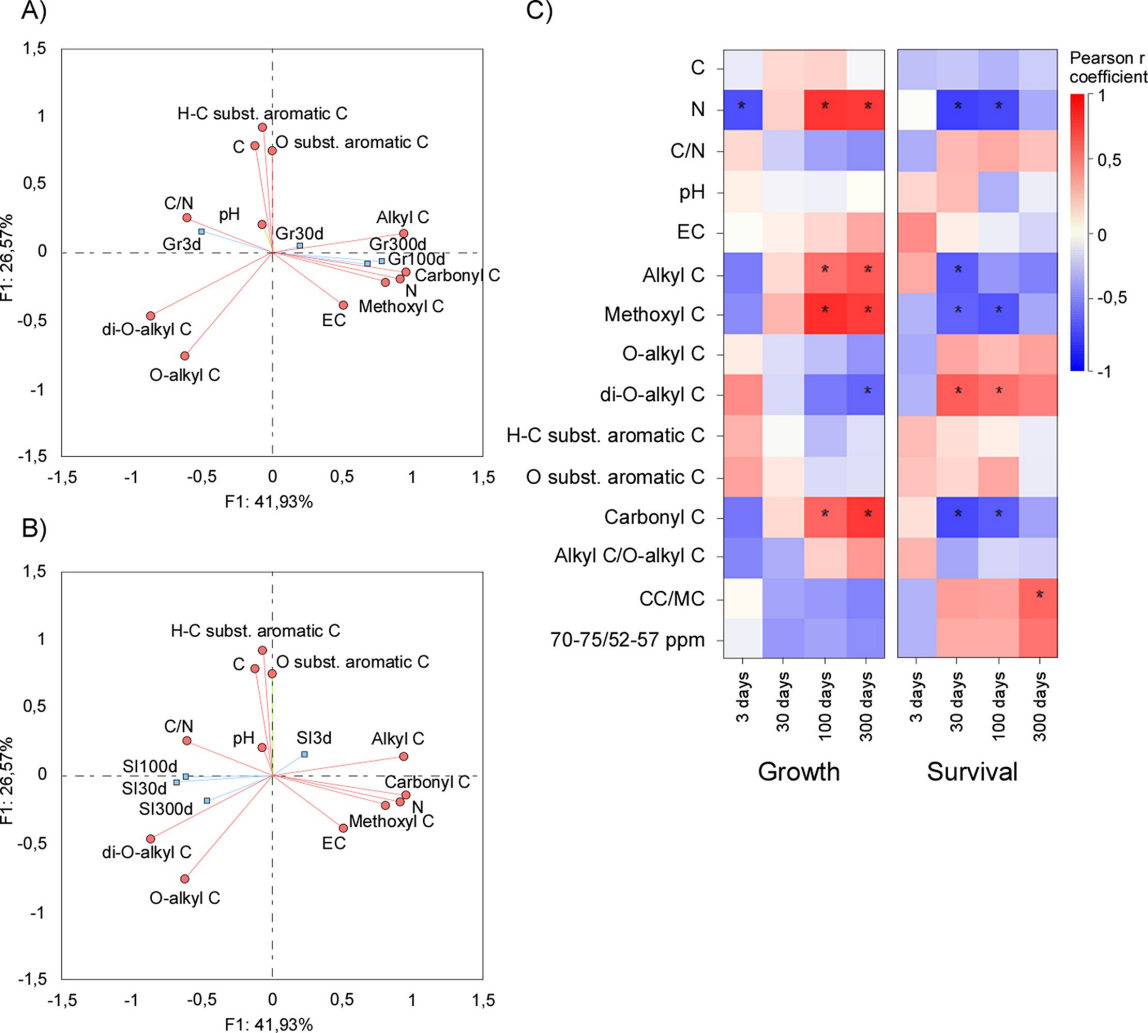
Correlation between growth and survival index (SI) of *Lactuca sativa* and chemical quality of the organic materials used for the soil amendment. (A) PCA biplot of the reference spectral regions and the chemical composition in the growth (A) and in the survival index (B) of *Lactuca sativa*. Data refer to loading vectors of the spectral regions and chemical characteristics (red vectors) and factorial scores of growth and survival index (blue vectors). (C) Heat-map correlating the growth and survival of *Lactuca sativa* target organisms with the chemical parameters of the 14 organic amendments used in the bioassays. Asterisks indicate statistically significant correlation values.

*L*. *sativa* growth and disease suppression were variably associated with the chemical quality of OAs, depending considerably on the incubation time (Fig 5A, 5B and 5C). Principal component analysis (PCA) provided a satisfactory ordination of the OAs chemical parameters in relation to the *L*. *sativa* growth and survival index (eigenvalues of the first two components accounting for 68.50% of the total variance—41.93% for first and 26.57% for the second component). In addition, PCA highlighted that the association between OAs chemistry with plant growth and disease suppression largely shifted during incubation time, both for ^13^C CPMAS NMR derived parameters and elemental chemical features.

Concerning plant growth, the first and second PCA components highlight the importance of C/N ratio, N content, and several NMR regions to explain the influence of OAs chemistry. Heat maps correlation confirmed that initial opposite and changing role of N content and C/N of OA, while pH and EC showed a general trend of no significant correlation. In detail, N content was negatively associated in short-term (3 days), to become strongly positive in the later decay phases (100 and 300 days). A similar pattern was found for alkyl C, methoxyl C, and carbonyl C regions (Fig 5C). An opposite pattern, with a shift from positive to negative correlation from early to late decay phase, was found for C/N ratio, O-alkyl C and di-O-alkyl C fractions.

PCA and correlation analyses clarified the limited role of pH, EC and C content in explaining diseases suppression. More important, our analyses highlight that the association between chemical parameters and disease suppression shifted during decomposition, oppositely to the trends described for the plant growth. In fact, C/N ratio, O-alkyl and especially di O-alkyl C fraction were negatively related in the short-term (3 days), but shifted to positive after 100 and 300 days of decomposition. On the contrary, N content, alkyl C, methoxyl C, and carbonyl C fractions showed significant positive correlation only at the intermediate dates of decomposition (30 and 100 days). Finally, the alkyl C / O-alkyl C and CC / MC ratio were generally unrelated to disease suppression, with the only exception of a positive correlation with CC / MC after 300 days of incubation.

## 4. Discussion

Our experiment, based on 14 different OAs with a broad range of chemistry and incubation periods ranging from 3 to 300 days, demonstrated that feedstock type and their decomposition affect significantly plant growth and disease suppression. As general trends, we recorded that N rich OAs with high decomposability were conducive in short-term, becoming more effective in suppressing *R*. *solani* and promoting plant growth if in decomposed state. On the other hand, cellulose-rich OAs with high C/N ratio were less capable of promoting *L*. *sativa* growth but were more suppressive to *R*. *solani*. Our study, for the first time, highlighting a trade-off between plant growth promotion and disease control capability modulated by OA decomposition stage. The trade-off would be considered for an effective OA application and integration in agricultural practices, trying to identify the conditions that optimize the balance between disease suppression and crop yield.

### 4.1. Organic amendments and disease suppression dynamics

Suppression of *R*. *solani* disease following OAs has been previously reported, but also the opposite effect with disease enhancment was found [25,26,27,36]. Accordingly, in our experiment disease suppression dynamics were highly variable among the OAs and over the 300 days of incubation, with suppressive effects recorded in 48% of the cases (27 observations over 56 OA combinations).

Decomposition process continuously modifies OAs chemistry and associated microbiome, thus affecting disease suppression [37,38]. The meta-analysis of Bonanomi et al. [39] reported that OA decomposition had a significant effect in 73% of the studies analyzed (*N* = 426). Here, decomposition affected disease suppression in 92% of OAs types, with a consistent disease suppression reported only for humus. We found that OAs response to decomposition could be categorized into four main groups: the first includes only humus that was consistently suppressive, the second group is made of N rich OAs (i.e. meat powder and fish meal) that were conducive in the short (days) and medium (weeks) term, but suppressive thereafter, the third group (compost pam, sawdust, cellulose, coconut fiber, peat, and biochar) showed a trend of increasing suppression with decomposition time. Instead, the last group, that include glucose, plant litter (*M*. *sativa* and maize) and FORSU, showed a more variable response to disease suppression during decomposition.

Humus extract was the only OA that *in vitro* completely inhibited *R*. *solani* growth, suggesting that the observed disease suppression is due to direct inhibition of the pathogen. Indeed, humus extract was the most suppressive OA and the only one with a consistent disease suppression during decomposition. This OA has a unique chemical composition that apart from being rich of organic N with a low C/N ratio, it is as well rich of aromatic compounds coupled with low content of di-O-alkyl C and O-alkyl C fractions related to sugar and easily microbial accessible polysaccharides. Usually, microbes including *R*. *solani*, thrived on OAs rich in sugars labile C fractions, and showed weak growth on materials rich in lignin and aromatic C fractions like humus [7]. From this point of view, humus extract seems to be the best OA for suppression of *R*. *solani*, but without pathogen inoculum, *L*. *sativa* was consistently inhibited by this material at the tested application rate. In fact, humus at high concentration was phytotoxic, indicating the importance of optimizing the application rate to combine plant growth promotion and disease suppression.

Differently from humus, OAs rich in N and labile organic carbon (meat powder and fish meal) when incorporated in soil are subjected to rapid and deep changes in a time frame of hours and days, with a rapid release of mineral nitrogen [40] and positive impact on soil aggregation [41]. Several studies demonstrated that the short-term and temporary accumulation of ammonia following meat and fish meal application can suppress the disease caused by *V*. *dahliae* by killing their microsclerotia [8]. Here, instead of that, the OAs were largely conducive for the damping-off in the short and medium-term, with a slight suppressive effect only after 300 days of incubation. *R*. *solani*, compared to other pathogens (e.g. *Pythium*, *Verticilium*, *Thielaviopsis*) is a polyphagous and strong saprophyte capable to exploit a range of OAs types [17,42]. Our results suggest that *R*. *solani* could, in the short-term, profit of the large availability of organic N and labile carbon released by meat and fish meal, causing a dramatic increase of damping-off incidence. Further data about *R*. *solani* population following OAs application are needed to test this hypothesis. In addition, in the short-term the phytotoxic effect of such OAs could synergistically interact with *R*. *solani*, enhancing damping-off incidence [43]. Our data, according with previous studies [7,44], indicate that planting after meat and fish meal application must be delayed at least 100 days to avoid direct phytotoxicity and an increased incidence of damping-off. In more genereral terms, the typical “toxic” time lag of crop residues and OAs range from few days to several weeks when soil temperature and moisture are within the range for optimal microbial decomposition. In this context, the use of ^13^C NMR is very useful to identify the state of biological maturation of OA and the reduction of phytotoxicity that is associated with progressive decrease in O-alkyl-C coupled with a relative increase of the alkyl-C region substrates [21, 28]. However, in the long-term when phytotoxicity disapper, the positive effects of these OAs due to N mineralization and improved soil structure become evident, with these materials inducing the most intense plant growth promotion of *L*. *sativa*. Then, meat and fish meal appear very active as OAs, but must be managed carefully to gain the advantage and to avoid their side effects.

In our experiment, the group composed of compost pam, sawdust, cellulose, coconut fiber, peat, and biochar showed an increasing suppression with decomposition time. Within this group, cellulose sawdust, cellulose, coconut fiber, and peat are characterized by high C/N ratio and, as revealed by ^13^C CPMAS NMR, and have a large amount of sugars and cellulose. The lack of plant protection in the short-term could be explained by a possible increase of *R*. *solani* inoculum due to its cellulolytic activity. However, these OAs may stimulate the development of a competitive microbiome for limiting mineral N that, in the long-term, may impair the saprophytic activity of pathogens like *R*. *solani* [45] or *Fusarium solani* [46].

Despite the high C/N ratio, biochar does not induce short-term N immobilization [49] because of the recalcitrant nature of pyrogenic carbon that does not support microbe development [47]. However, in the long-term the observed disease suppression could be related to the development of a suppressive microbiome thank to the porous structure of biochar that protects bacteria and fungi from grazers and abiotic stress like drought [48]. In general, the large variability of biochar chemistry associated with the initial feedstock and pyrolysis conditions (i.e. oxygen availability, temperature, and duration of thermal treatment), must be taken into account to understand the suppressive capability of biochar.

Disease suppression after amendment with maize litter and glucose showed a typical Ո-shaped dynamic. On the other hand, we found that glucose, cellulose, grass litter and sawdust immobilized soil N, with a strongly dependent effect on incubation time. Maize litter is rich with sugars and cellulose but poor of N, while glucose is readily consumed by the majority of soil microbes. As a result, these OAs induces a rapid, but short-term N immobilization [49]. Then, after 3 days of incubation, *R*. *solani* could take advantage of the available carbon source for growth, causing a severe damping-off. Thereafter, the N immobilization would likely limit *R*. *solani* proliferation and the associated damping-off [17]. Finally, in the later stage (300 days) the rapid microbial turnover probably releases back the immobilized N into the soil [50], supporting the activity of the pathogen. This complex pattern of N immobilization and mineralization is consistent with U-shaped response of *L*. *sativa* growth in absence of *R*. *solani* inoculum. In this case, N immobilization explains well the growth inhibition observed in the intermediate phase of decomposition (30 and 100 days). Although microbial activity and *R*. *solani* population were not measured, we suspect that specular pattern of disease suppression and plant growth promotion is well explained by the dynamics of mineral N. Further studies, however, are required to confirm this speculation.

Finally, *Medicago* litter and FORSU were the only OAs showing a U-shaped dynamic of disease suppression. The short-term suppression cannot be explained with a direct inhibitory effect because *R*. *solani* readily uses these OAs *in vitro* conditions. On the other hand, it is possible that *R*. *solani* uses such OAs as a substrate for inoculum built-up in the medium term, causing the observed increase in damping-off incidence. In general, the relationship between the decomposition age of crop residues and disease suppression is quite variable and still difficult to be explained. This study also highlights the importance of future research to reveal the complex linkage between soil microbiome, OAs chemistry, and disease suppression.

### 4.2. In search of chemical predictors of disease suppression

The identification of reliable indicators of OAs suppressive capability is of paramount importance for rationale management of this practice in agroecosystems. In fact, if OA is determined as suppressive to some pathogens and conducive to others or acts differently to the environmental conditions, it cannot be effectively applied by farmers for the control of plant diseases. In this context, predicting *R*. *solani* suppression by OAs is notoriously difficult using chemical, enzymatic, and microbiological parameters [26,27]. Bonanomi et al. [39] in a meta-analysis based on 81 parameters and 643 correlations concluded that OAs chemical parameters are unable to predict disease suppression towards *R*. *solani*. Accordingly, in this study C/N ratio, pH and EC showed a prevalence of insignificant correlations despite the wide range of values reported for these parameters. C/N ratio is a common indicator of OAs chemical characteristics, and previously used to predict their agronomic impact. However, recent studies demonstrate the poor capability of this parameter to predict soil functions like aggregate stability, N mineralization and litter decomposition [49]. The greatest weakness of the C/N ratio lies in the fact that it refers only to the total amount of organic carbon, not defining its chemical composition, which could range from sugars to aromatic fractions as occur in lignin-rich OAs or biochar.

Differently, we found that OAs chemistry defined by ^13^C-CPMAS NMR provide useful insight to predict disease suppression. In particular, the di-*O*-alkyl C fraction was positively associated with disease suppression at medium-to-long term. For instance, Kadvir et al [50] reported that the carbohydrate content of OAs was positively correlated with soil activity and with the stability of soil aggregates. Interestingly, significant negative correlations with disease suppression were found for aliphatic, methoxyl and carboxyl C fractions in the medium-term observations. A similar pattern was found for total N content of OAs. Such result mainly depends on the high content of total N and these molecular fractions in meat powder, fish meal and, to a lesser extent, *Medicago* litter. In soil samples incubated with such OAs, the increase of damping-off incidence suggests a rapid but transient burst of *R*. *solani* activity that takes advantage of polysaccharide and N compounds that are rapidly degraded.

Most importantly, this study highlights that the correlation between disease suppression and N content or NMR spectral regions substantially changed during the incubation time. For instance, an OA with a high abundance of carbonyl C, methoxyl C, and total N would be conducive to damping-off in the short-terms but suppressive after 300 days of incubation. This shift in correlation between chemical parameters and disease suppression likely reflect the changes in soil chemistry and microbiology that occur during decomposition. Taking into account the variable significance of each parameter with decomposition time is pivotal for an effective prediction of OAs suppressiveness. Our data are based on extensive correlative analysis (56 combinations of OAs types and decomposition time), but is only an early step to fully appreciate the implications of OAs use for agronomic purposes. Further works may refine the information obtainable by regions of the ^13^C-CPMAS NMR with other microbial and functional parameters like soil respiration, and enzymatic activities.

## 5. Conclusion

Effective and rational management of OAs in agroecosystems must be based on well-characterized materials having positive effects on soil functioning, crop productivity, and disease suppression. This study systematically explored the interaction between OAs types and decomposition time, demonstrating that most of the organic materials were either suppressive or conducive to damping-off in relation to feedstock origin and incubation time. Then, the idea that some OAs are suppressive and others are conducive must be overcome, facing with the shift in disease suppressiveness observed during decomposition. Moreover, our results provide evidence that, for several OAs, plant growth promotion was negative correlated with disease suppression, highlighting a trade-off between these agro-ecosystem functions. With this in mind, it is necessary to identify the OAs types and application timing that optimize the beneficial effects of the amendment, limiting their side effects i.e. phytotoxicity, N immobilization that impair root proliferation, damping-off conduciveness. In addition, we found that the effects of OAs can be hardly predictable based on the chemical characteristic, although N content and some ^13^C CPMAS NMR regions (alkyl C, methoxyl C, and carbonyl C) showed significant correlation with disease suppression. This, therefore, could provide an advance in the prediction of OAs suppressiveness. However, it is still evident that no chemical variable can be used alone as a reliable predictor of disease suppression, especially if decomposition time is not taken under consideration.

We are aware that our experiment was based on a limited number of OAs and only one soil type. Moreover, this study was carried out under optimal conditions of temperature and soil moisture regime, and then further research should investigate the consistency of finding under more variable conditions. Finally, in the present work, we focus on chemical parameters to characterize OAs, overlooking the impact on soil microbiome taxonomy, and functions on disease suppression. In fact, when applied to the soil OAs profoundly modify all living components with cascade effects on soil functioning. Future studies are needed to understand the effect of different OAs types and decomposition time on different microbial trophic group to improve our understanding of disease suppression.

## Supporting information

S1 Table Results of two-way analysis of variance of root growth for *Lepidium sativum* and *Lactuca sativa* treated with aqueous extract from different organic amendments at different concentrations. Significance level fixed at p-values < 0.05.
(DOCX)

S2 Table Results of analysis of variance of growth of the fungus *Rhizoctonia solani* treated with different water extracts organic amendments. Significance level fixed at p-values < 0.05.
(DOCX)

S3 Table Results of two-way analysis of variance of growth of *Lactuca sativa* treated with different organic amendments at different time of decomposition. Significance level fixed at p-values < 0.05.
(DOCX)

S4 Table Results of two-way analysis of variance of survival index of *Lactuca sativa* treated with different organic amendments at different day of decomposition. Significance level fixed at p-values < 0.05.
(DOCX)
