# Supplementary material for: Decomposition and organic amendments chemistry explain contrasting effects on plant growth promotion and suppression of Rhizoctonia solani damping off
Source: PLoS One. 2020 Apr 9;15(4):e0230925. doi: 10.1371/journal.pone.0230925 (PMC7144968; doi:10.1371/journal.pone.0230925)
Supplement: S1 Table — Significance level fixed at p-values < 0.05. (DOCX) [file pone.0230925.s001.docx]

**S1 Table.**

|  | *L. sativum* | | *L. sativa* | |
| --- | --- | --- | --- | --- |
|  | *F* | *p-value* | *F* | *p-value* |
| Intercept | 2744.139 | **<0.001** | 1361.037 | **<0.001** |
| Organic Amendments (OAs) | 14.370 | **<0.001** | 10.232 | **<0.001** |
| Extract Concentrations (ECs) | 47.047 | **<0.001** | 14.236 | **<0.001** |
| OAs × ECs | 6.089 | **<0.001** | 3.664 | **<0.001** |
