# Supplementary material for: Decomposition and organic amendments chemistry explain contrasting effects on plant growth promotion and suppression of Rhizoctonia solani damping off
Source: PLoS One. 2020 Apr 9;15(4):e0230925. doi: 10.1371/journal.pone.0230925 (PMC7144968; doi:10.1371/journal.pone.0230925)
Supplement: S2 Table — Significance level fixed at p-values < 0.05. (DOCX) [file pone.0230925.s002.docx]

**S2 Table.**

|  | *SS* | *DF* | *MS* | *F* | *p-value* |
| --- | --- | --- | --- | --- | --- |
| Intercept | 2.295236E+10 | 1 | 2.295236E+10 | 558.5705 | **<0.001** |
| Organic Amendments | 7.779327E+09 | 13 | 5.984097E+08 | 14.5629 | **<0.001** |
