# Supplementary material for: Decomposition and organic amendments chemistry explain contrasting effects on plant growth promotion and suppression of Rhizoctonia solani damping off
Source: PLoS One. 2020 Apr 9;15(4):e0230925. doi: 10.1371/journal.pone.0230925 (PMC7144968; doi:10.1371/journal.pone.0230925)
Supplement: S3 Table — Significance level fixed at p-values < 0.05. (DOCX) [file pone.0230925.s003.docx]

**S3 Table**

|  | *SS* | *DF* | *MS* | *F* | *p-value* |
| --- | --- | --- | --- | --- | --- |
| Intercept | 6.89703 | 1 | 6.897034 | 211.8630 | **<0.001** |
| Day of decomposition (DD) | 1.99317 | 3 | 0.664390 | 20.4087 | **<0.001** |
| Organic Amendments (OAs) | 2.78321 | 14 | 0.198801 | 6.1068 | **<0.001** |
| DD × OAs | 4.12647 | 42 | 0.098249 | 3.0180 | **<0.001** |
