# Supplementary material for: Decomposition and organic amendments chemistry explain contrasting effects on plant growth promotion and suppression of Rhizoctonia solani damping off
Source: PLoS One. 2020 Apr 9;15(4):e0230925. doi: 10.1371/journal.pone.0230925 (PMC7144968; doi:10.1371/journal.pone.0230925)
Supplement: S4 Table — Significance level fixed at p-values < 0.05. (DOCX) [file pone.0230925.s004.docx]

**S4 Table.**

|  | *SS* | *DF* | *MS* | *F* | *p-value* |
| --- | --- | --- | --- | --- | --- |
| Intercept | 23610.96 | 1 | 23610.96 | 741.5154 | **<0.001** |
| Day of Decomposition (DD) | 7731.20 | 3 | 2577.07 | 80.9342 | **<0.001** |
| Organic Amendments (OAs) | 4038.74 | 13 | 310.67 | 9.7568 | **<0.001** |
| DD × OAs | 4844.20 | 39 | 124.21 | 3.9009 | **<0.001** |
